# Association Between Mean Left Atrial Voltage and Prior Ischemic Stroke in Atrial Fibrillation: A Single-Center Cross-Sectional Study

**DOI:** 10.3390/jcm15187036

**Published:** 2026-09-11

**Authors:** Ha Young Yu, Chang-Ok Seo, Yun Gi Kim, Jong-Il Choi, Joo Hee Jeong, Jaemin Shim, Seong-Mi Park, Young-Hoon Kim

**Affiliations:** Division of Cardiology, Department of Internal Medicine, Korea University College of Medicine and Korea University Anam Hospital, 73 Goryeodae-ro, Seongbuk-gu, Seoul 02841, Republic of Korea; rhy707@gmail.com (H.Y.Y.); changokseo@gmail.com (C.-O.S.); jessica0115@naver.com (J.H.J.); jaemins@korea.ac.kr (J.S.); smparkmd@korea.ac.kr (S.-M.P.); yhkmd@korea.ac.kr (Y.-H.K.)

**Keywords:** atrial fibrillation, left atrial voltage, ischemic stroke, atrial cardiomyopathy, low-voltage area

## Abstract

**Background/Objectives:** Left atrial (LA) low-voltage area quantification is time-consuming and threshold-dependent. We investigated whether mean left atrial bipolar voltage, as a marker of atrial cardiomyopathy, is associated with prior ischemic stroke and compared its performance with low-voltage area percentage (LVA%). **Methods:** In this single-center retrospective cross-sectional study, 259 consecutive patients with atrial fibrillation (AF) undergoing radiofrequency catheter ablation were enrolled. Mean LA voltage was calculated as the arithmetic mean of all bipolar voltage points acquired during electroanatomic mapping. LVA% was defined as the proportion of LA surface area with voltage < 0.5 mV. Associations with prior ischemic stroke were assessed using Firth’s penalized logistic regression. **Results:** The median age was 67 years and 31.7% were women. Twenty-nine patients (11.2%) had prior ischemic stroke. Patients with prior stroke had significantly lower mean LA voltage (0.5 [0.4–0.8] vs. 1.0 [0.6–1.3] mV, *p <* 0.001). After adjustment for CHA_2_DS_2_-VASc components, mean LA voltage remained independently associated with prior stroke (odds ratio 0.12, 95% confidence interval 0.03–0.38; *p <* 0.001). Mean LA voltage and LVA% showed comparable discrimination (area under the curve 0.743 vs. 0.717; *p =* 0.392), but adding mean LA voltage to the LVA%-only model improved model fit (likelihood ratio test *p* = 0.024). **Conclusions:** In this cross-sectional cohort, lower mean LA voltage was independently associated with prior ischemic stroke and may better reflect the extent of underlying atrial remodeling than LVA%.

## 1. Introduction

Ischemic stroke is a major complication of atrial fibrillation (AF) [1,2]. Various risk factors for ischemic stroke in AF patients have been reported, including age, sex, hypertension, diabetes mellitus, heart failure, prior ischemic stroke, or vascular disease [3,4]. These risk factors are incorporated into the CHA_2_DS_2_-VASc scoring system which has been widely adopted to predict stroke risk and to guide anticoagulation treatment [3,5,6]. In addition to the CHA_2_DS_2_-VASc score and its individual components, several other important risk factors have been identified such as morphology of the left atrial appendage (LAA), N-terminal B-type natriuretic peptide (NT-proBNP), cardiac troponin, spontaneous echo contrast in LAA, and enlargement of the left atrium (LA) [7,8,9,10]. Bipolar low-voltage areas (LVAs) in the LA have also been identified as a risk factor for ischemic stroke in AF patients [11,12].

Acquiring bipolar voltage of the LA can vary significantly depending on the type of mapping catheter, acquisition program, methods for selecting signals to be measured, appropriate catheter contact, and operator experience. Most studies calculated LVA by summing all low-voltage surface areas and then dividing by the total mapped surface area of the LA. However, this method requires a large amount of time and effort to acquire LVA. Furthermore, evidence for applying a certain cut-off value (<0.5 mV) for defining low-voltage is not concrete. A prior study demonstrated that the presence of LVA is associated with a diffuse voltage reduction in the LA, indicating atrial cardiomyopathy is a global phenomenon affecting the entire LA rather than specific sites [13]. To avoid such limitations, we attempted to simplify the quantification of atrial remodeling by calculating the mean value of all acquired bipolar voltage points of the LA. The purpose of this study was to confirm whether our method to quantify the degree of atrial cardiomyopathy, which is mean LA voltage, is associated with prior ischemic stroke in patients with AF undergoing first ablation. Furthermore, we aimed to compare its discriminative performance with that of LVA, which has traditionally been used as an electroanatomical surrogate of atrial remodeling.

## 2. Materials and Methods

### 2.1. Patients

This was a single-center, retrospective observational study. We enrolled consecutive AF patients undergoing first radiofrequency catheter ablation (RFCA) at Korea University Medical Center Anam Hospital by a single operator (Yun Gi Kim) between November 2024 and November 2025. There were no exclusion criteria. The Institutional Review Board of Korea University Medical Center Anam Hospital approved this study (IRB No. 2025AN0289) and waived written informed consent because the current study was a retrospective analysis. We strictly adhered to the ethical guidelines of the 2013 Declaration of Helsinki and legal regulations of South Korea.

### 2.2. Electroanatomic Mapping and Voltage Acquisition

Electroanatomic mapping and ablation were performed following the RFCA protocol of our institution, which has been reported in our previous studies [14,15,16]. Before trans-septal puncture, duo-decapolar and decapolar catheters were positioned to record electrograms of the high right atrium, low right atrium, coronary sinus ostium, and distal coronary sinus. Two trans-septal punctures were performed. The EnSite X system (Abbott, Chicago, IL, USA) was used to perform three-dimensional electroanatomic mapping. Mapping was done during sinus rhythm. If the initial rhythm was AF, internal cardioversion using duo-decapolar and decapolar catheters was performed. Whenever immediate triggers of AF were observed, repeat internal cardioversion was performed up to 3 attempts. If sinus rhythm was not achievable despite repeat internal cardioversions, electroanatomic mapping was done during AF. The variable-loop circular mapping catheter (Abbott, Chicago, IL, USA) was used for three-dimensional mapping and bipolar voltage acquisition. Bipolar voltage was defined as the amplitude of the peak-to-peak electrogram of each selected electrogram. A peak frequency algorithm was applied to determine which bipolar voltages were recorded. Prior studies reported that the association between peak frequency and maximal peak-to-peak voltage is weak [17]. Recent reports suggest that the peak frequency acquisition method may help to characterize a tachycardia circuit by selectively acquiring near-field potentials rather than far-field potentials [18]. Only the LA body was used for analysis; areas beyond the pulmonary vein ostia and LAA were not included. The mapping process was done in a standardized order to minimize patient-to-patient variation. It started at the left inferior pulmonary vein, followed by the left superior pulmonary vein, high posterior wall, roof, and right superior and inferior pulmonary vein. After acquiring all pulmonary veins, the low septum and low anterior wall were mapped by looping of the mapping catheter. By slowly releasing the curve of the mapping catheter, the low posterior wall and perimitral isthmus area were mapped. By further release and advancement, the mapping catheter was inserted into the LA appendage. The anterior wall of the LA was then mapped which was followed by high septum mapping.

For LA voltage evaluation, the individual value of all mapped points was extracted from the EnSite X system and analyzed. During voltage mapping, two elements were measured: (i) mean voltage of the LA, which was the arithmetic mean of all bipolar voltages acquired during the mapping process; (ii) LVA%, which was calculated by ([area under 0.5 mV/LA surface area] × 100).

### 2.3. Definition of Prior Ischemic Stroke

Prior ischemic stroke was defined as a neurologist-confirmed diagnosis of ischemic stroke documented in the medical record before the index ablation procedure. All events were confirmed by neuroimaging (computed tomography or magnetic resonance imaging) and were ascertained through systematic review of electronic medical records. Transient ischemic attack was not included. Clinical characteristics, laboratory data, and echocardiographic parameters were extracted from the institutional database.

### 2.4. Imaging Evaluation

Computed tomography (CT) was performed to exclude the presence of thrombus in the LAA. The diameter of the LA and systolic function of the left ventricle were measured with transthoracic echocardiography performed prior to the RFCA procedure.

### 2.5. Statistical Analysis

Continuous variables are presented as means with standard deviation (SD) or medians with interquartile ranges (IQRs), as appropriate, and categorical variables as frequencies and percentages. Group comparisons were performed using appropriate statistical tests according to data distribution. Associations between mean LA voltage, LVA%, and other clinical variables were assessed using Spearman rank correlation analysis. To further evaluate potential nonlinear relationships between mean LA voltage and LVA%, restricted cubic spline regression models with four knots were constructed. The overall and nonlinear components were tested using analysis of variance. Univariable and multivariable logistic regression analyses were performed using Firth’s penalized likelihood method to address potential separation and small event numbers. The multivariable model included the components of the CHA_2_DS_2_-VASc score—age, sex, hypertension, diabetes mellitus, heart failure, and vascular disease—together with mean LA voltage, as these represent established and clinically validated risk factors for thromboembolism in AF. LVA% was not included in the multivariable model because of its strong collinearity with mean LA voltage. For sensitivity analyses, we additionally adjusted the multivariable model for mapping rhythm (AF vs. sinus rhythm during mapping) and the number of mapped points to account for potential measurement-related confounding, and repeated the model in the subgroup mapped during sinus rhythm. Discriminative performance of mean LA voltage and LVA% for ischemic stroke was evaluated using receiver operating characteristic (ROC) curve analysis, and the area under the ROC curve (AUC) was calculated. Differences between correlated ROC curves were assessed using a bootstrap method. Model performance was further compared using the Akaike information criterion (AIC), Nagelkerke’s pseudo-R^2^, and likelihood ratio tests for nested models to evaluate the incremental explanatory value. A two-sided *p* value < 0.05 was considered statistically significant. Missing data were handled using complete-case analysis, and no imputation was performed. Statistical analyses were performed in R (version 4.4.3; R Foundation for Statistical Computing, Vienna, Austria).

## 3. Results

### 3.1. Patient Characteristics

**Figure 1:**
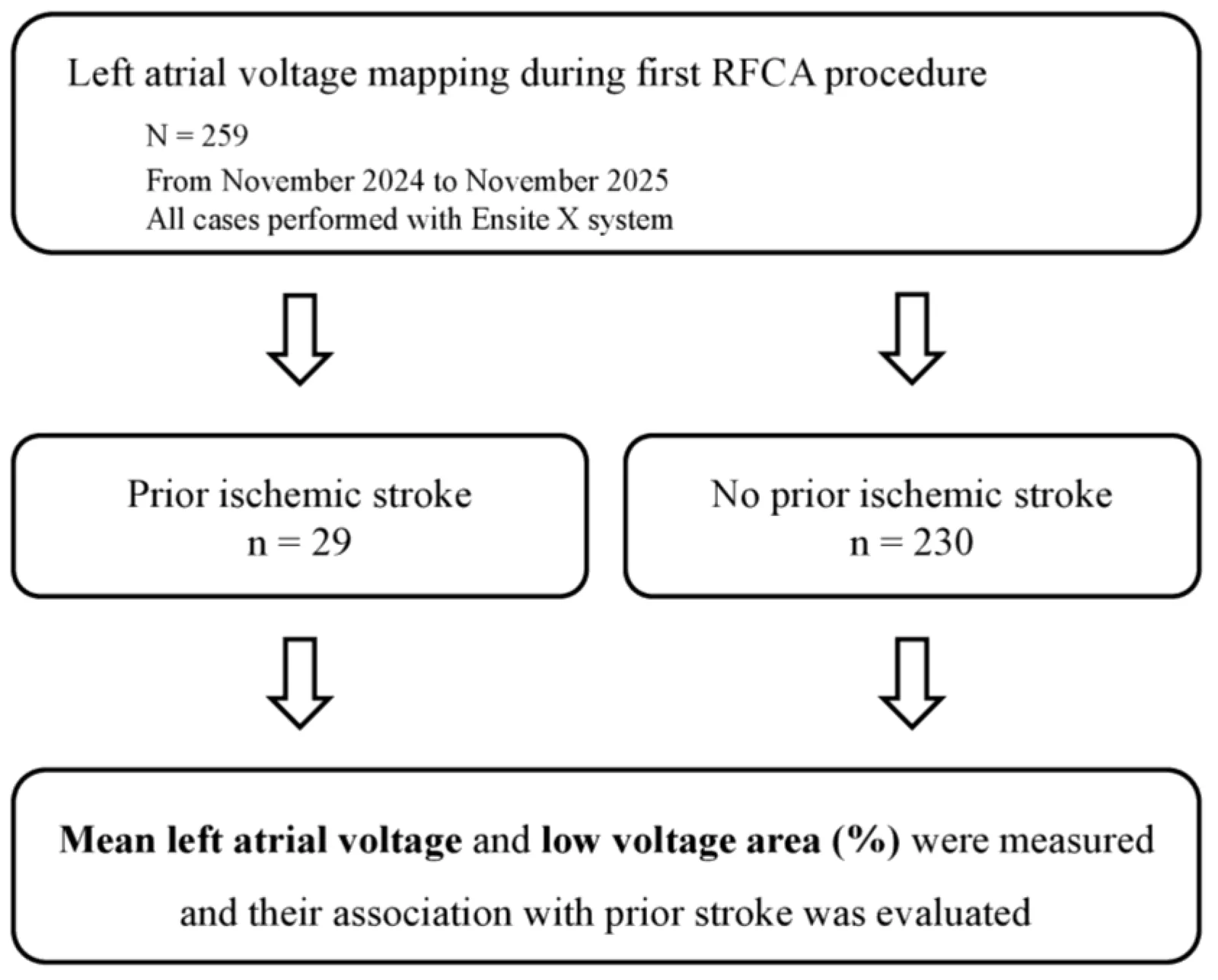
Flow of the study. Abbreviations: RFCA, radiofrequency catheter ablation.

A total of 259 patients were enrolled in the study (Figure 1). The median age was 67.0 years (interquartile range [IQR], 58.0–74.0), and 31.7% were women. A total of 29 patients (11.2%) had a prior history of ischemic stroke.

Among the 29 patients with prior ischemic stroke, the date of the index stroke was available in 22 (75.9%), with a median interval between the stroke and ablation of 7.9 months (IQR 5.6–46.4). The stroke preceded the diagnosis of AF in 8 patients (36.4%) and occurred at or after AF diagnosis in 14 (63.6%). Stroke subtype was classified as cardioembolic in 20 patients (69.0%) and as undetermined in 1 (3.4%), and could not be classified in 8 (27.6%). At the time of the index stroke, 25 patients (86.2%) were not receiving oral anticoagulation, 1 (3.4%) was receiving warfarin, and this information was unavailable in 3 (10.3%).

**Figure 2:**
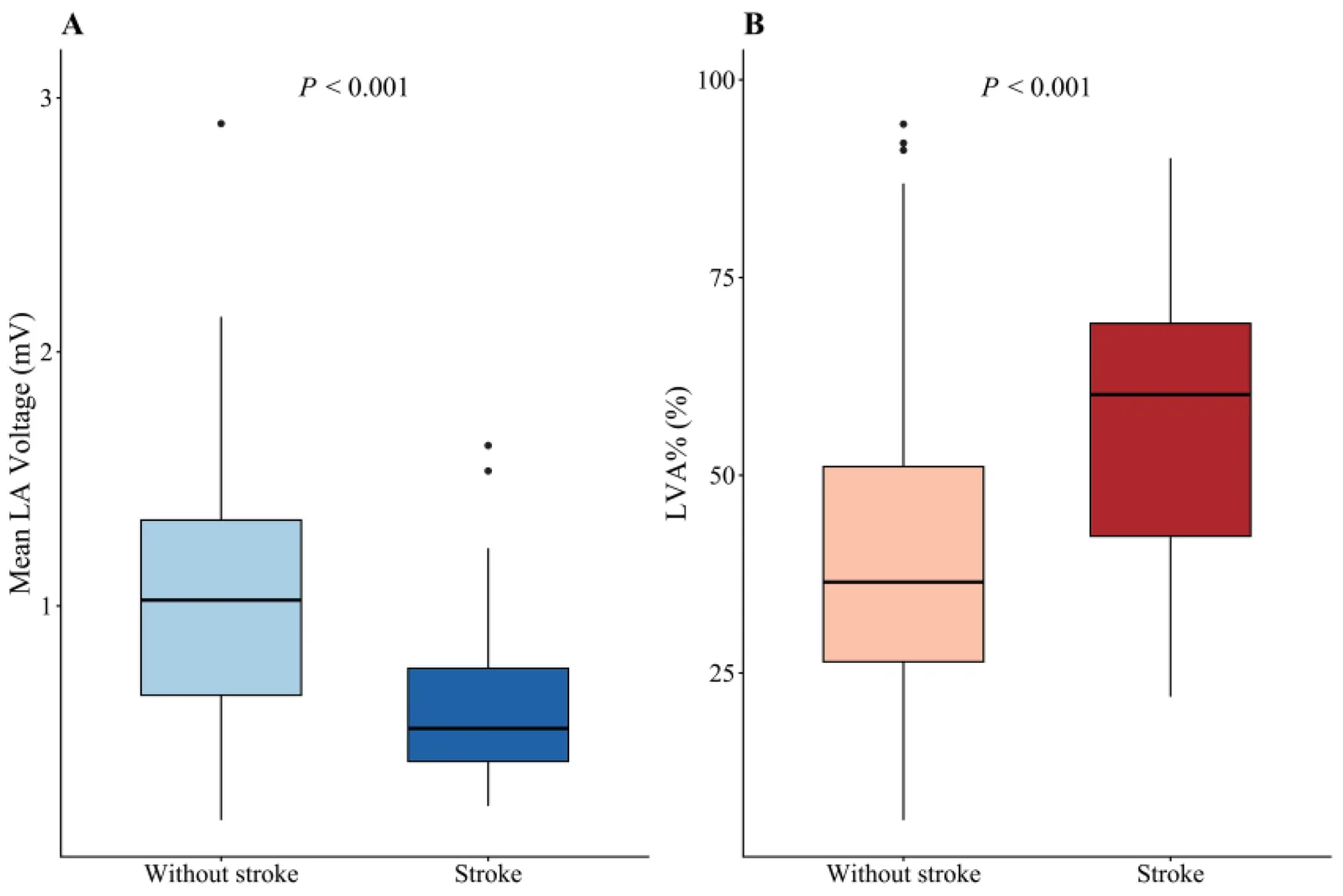
LA voltage in patients with and without prior ischemic stroke. Panel (**A**) shows that mean LA voltage was significantly lower in patients with prior ischemic stroke compared with those without stroke. Panel (**B**) shows that LVA% was significantly higher in patients with prior ischemic stroke. Abbreviations: LA, left atrial; LVA%, low-voltage area percentage.

Compared with patients without prior stroke, those with prior stroke were significantly older (72.0 [63.0–77.0] vs. 67.0 [58.0–74.0] years, *p =* 0.033) and had higher CHA_2_DS_2_-VASc scores (5.0 [3.0–5.0] vs. 2.0 [1.0–3.0], *p <* 0.001) (Table 1). Patients with prior stroke also exhibited more advanced atrial substrate remodeling, characterized by lower mean LA voltage (0.5 [0.4–0.8] vs. 1.0 [0.6–1.3] mV, *p <* 0.001; Figure 2A) and greater LVA% (60.2 [42.3–69.2] % vs. 36.5 [26.4–51.1] %, *p <* 0.001; Figure 2B).

### 3.2. Factors Associated with Prior Ischemic Stroke

In univariable Firth logistic regression analyses, older age (OR 1.05, 95% CI 1.01–1.09; *p =* 0.019), lower mean LA voltage (OR 0.11, 95% CI 0.03–0.32; *p <* 0.001), and higher LVA% (OR 1.04, 95% CI 1.02–1.06; *p <* 0.001) were significantly associated with prior ischemic stroke (Table 2). Other clinical variables, including sex, body mass index, hypertension, diabetes mellitus, heart failure, vascular disease, persistent AF, LA diameter, and NT-proBNP level, were not significantly associated with prior stroke in univariable analyses.

In the multivariable model including the components of the CHA_2_DS_2_-VASc score (age, sex, hypertension, diabetes mellitus, heart failure, and vascular disease) and mean LA voltage, only mean LA voltage remained independently associated with prior ischemic stroke (OR 0.12, 95% CI 0.03–0.38; *p <* 0.001). None of the conventional clinical risk factors was significantly associated with prior stroke in the multivariable analysis. These findings persisted after additional adjustment for the mapping rhythm and number of mapped points (OR 0.061, 95% CI 0.012–0.264; *p <* 0.001; Table S1). In an analysis restricted to the 232 patients mapped during sinus rhythm, mean LA voltage remained independently associated with prior ischemic stroke (OR 0.090, 95% CI 0.017–0.398; *p* = 0.001), with an effect estimate comparable to that of the overall cohort (Table S2).

### 3.3. Associations of Mean LA Voltage with LVA% and Clinical Variables

**Figure 3:**
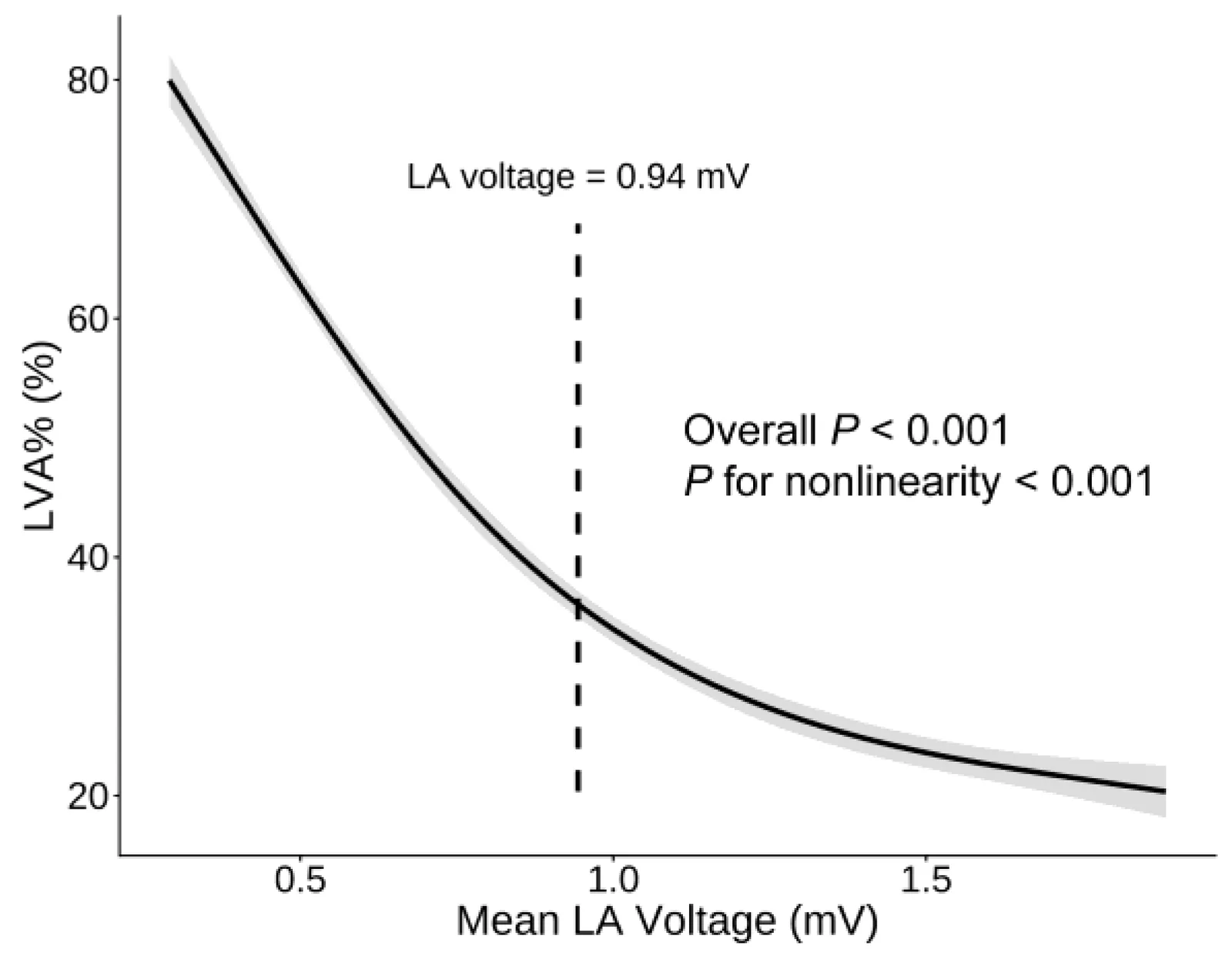
Nonlinear association between mean left atrial voltage and low-voltage area percentage. Restricted cubic spline analysis demonstrating the association between mean LA voltage and LVA%. The solid line represents the fitted spline curve and the shaded area indicates the 95% confidence interval. A significant inverse nonlinear association was observed (overall *p* < 0.001; *p* for nonlinearity < 0.001). Segmented regression analysis identified a breakpoint at 0.94 mV. Abbreviations: LA, left atrial; LVA%, low-voltage area percentage.

Spearman correlation analysis demonstrated a strong inverse correlation between mean LA voltage and LVA% (ρ = −0.93, *p <* 0.001). Restricted cubic spline analysis revealed a significant nonlinear association between mean LA voltage and LVA% (overall *p <* 0.001; nonlinearity *p <* 0.001) (Figure 3). A breakpoint was identified at 0.94 mV, below which LVA% decreased more steeply, whereas the decline was more gradual above this value.

**Figure 4:**
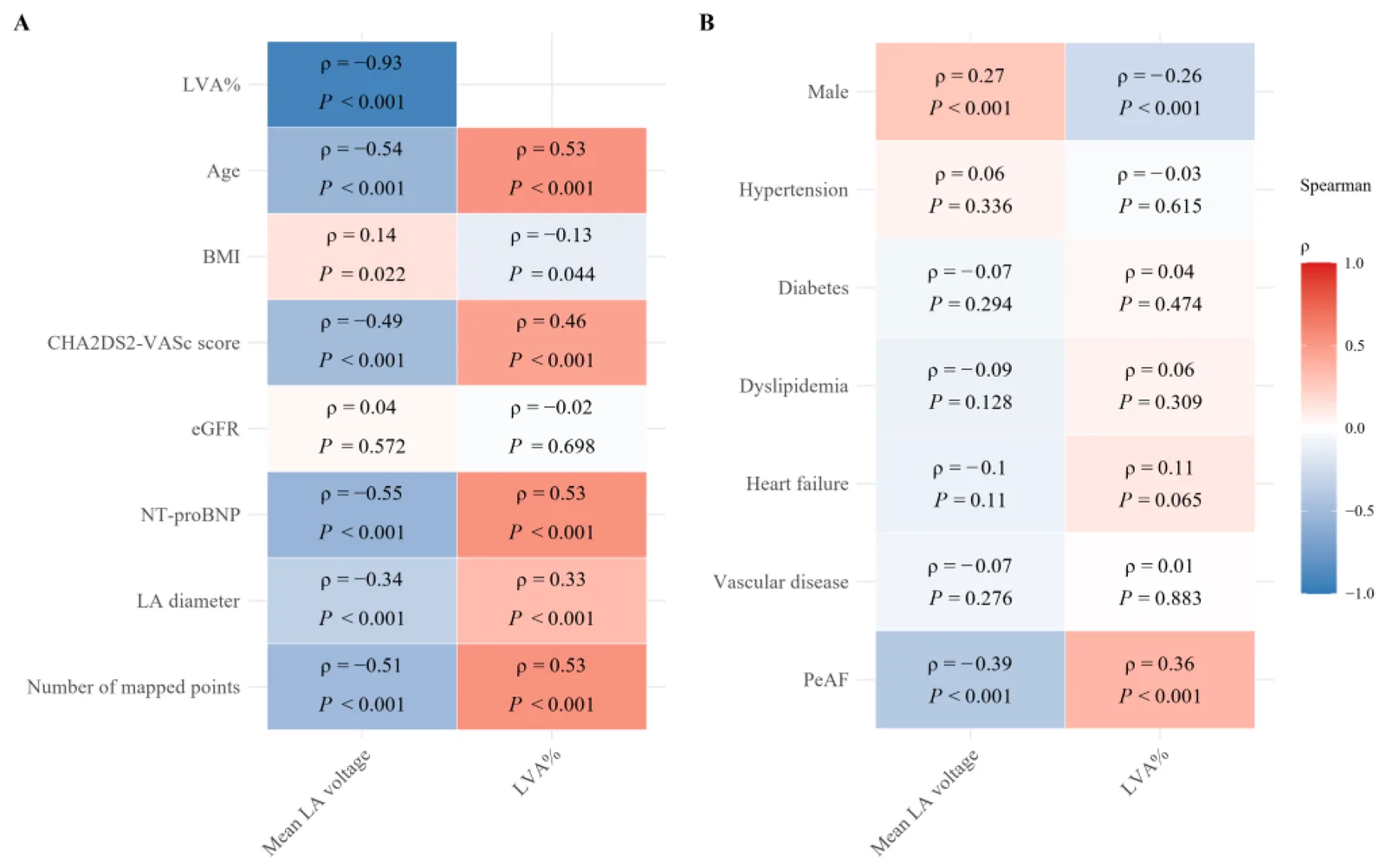
Spearman correlation heatmaps demonstrating associations between atrial substrate parameters and clinical variables. Panel (**A**) shows correlations among continuous variables and atrial substrate parameters. Panel (**B**) shows correlations between binary clinical characteristics and atrial substrate parameters. The color scale indicates the strength and direction of correlation. Correlation coefficients and corresponding p-values are displayed within each cell. Abbreviations: BMI, body mass index; eGFR, estimated glomerular filtration rate; LA, left atrium/left atrial; LVA%, low-voltage area percentage; NT-proBNP, N-terminal pro B-type natriuretic peptide; PeAF, persistent atrial fibrillation.

Mean LA voltage was significantly inversely correlated with age (ρ = −0.54, *p <* 0.001; Figure 4A), CHA_2_DS_2_-VASc score (ρ = −0.49, *p <* 0.001; Figure 4A), NT-proBNP (ρ = −0.55, *p <* 0.001; Figure 4A), LA diameter (ρ = −0.34, *p <* 0.001; Figure 4A), and number of mapped points (ρ = −0.51, *p <* 0.001; Figure 4A). In contrast, it showed a modest positive correlation with BMI (ρ = 0.14, *p =* 0.022; Figure 4A), while no significant correlation was observed with eGFR. For categorical variables, mean LA voltage was significantly higher in males (ρ = 0.27, *p <* 0.001; Figure 4B) and lower in patients with persistent AF (ρ = −0.39, *p <* 0.001; Figure 4B). Other clinical comorbidities, including hypertension, diabetes mellitus, dyslipidemia, heart failure, and vascular disease, were not significantly correlated with mean LA voltage. LVA% showed a similar but opposite correlation pattern.

### 3.4. Discriminative Performance of Mean LA Voltage and LVA%

**Figure 5:**
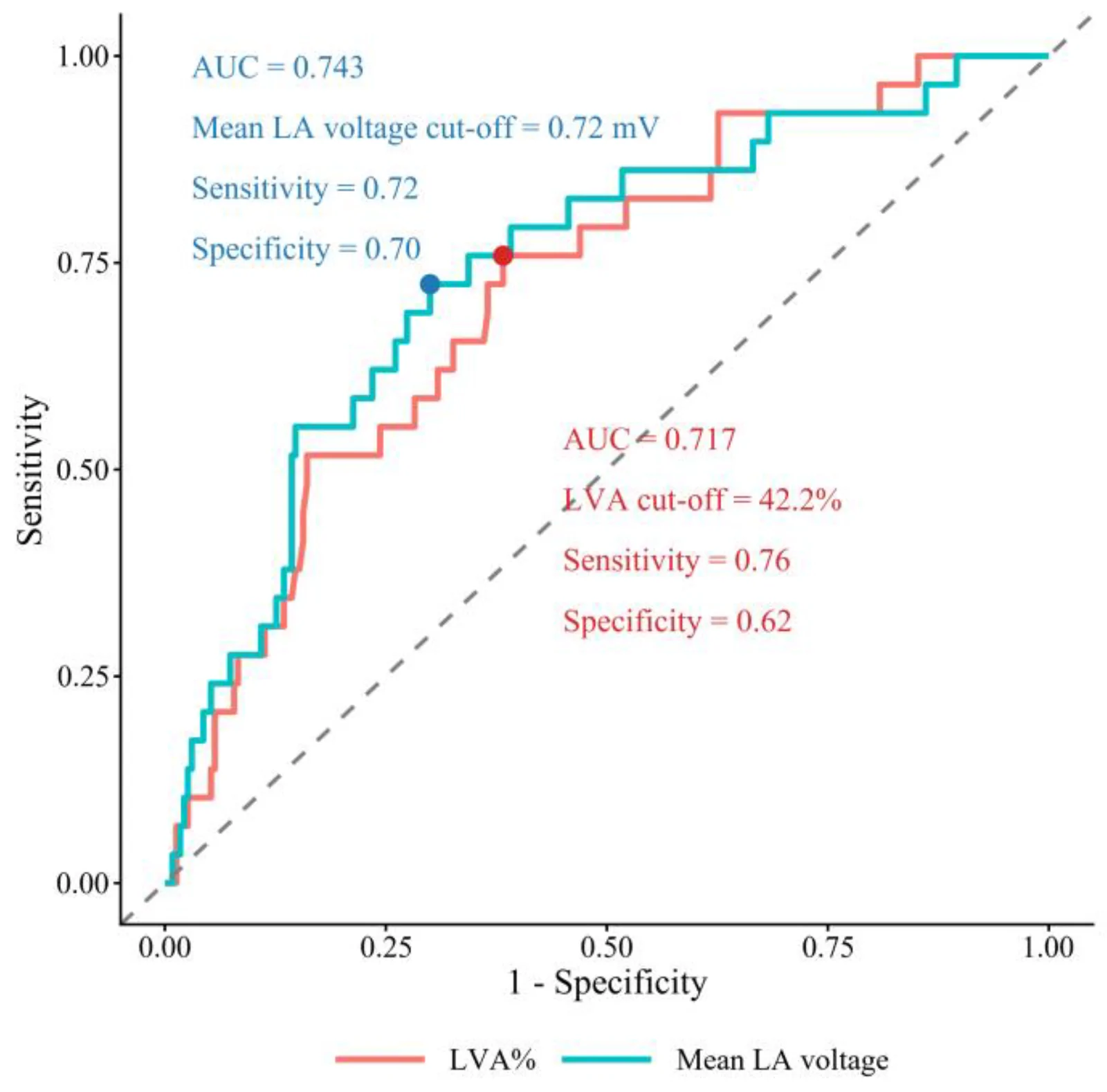
Receiver operating characteristic curves of mean left atrial voltage and low-voltage area percentage for discriminating prior ischemic stroke. Optimal cut-off values were determined using the Youden index. The blue and red dots indicate the optimal cut-off points for mean LA voltage and LVA%, respectively, and the dashed line represents the line of no discrimination. Abbreviations: AUC, area under the curve; LA, left atrial; LVA%, low-voltage area percentage; ROC, receiver operating characteristic.

Discriminative performance was comparable between the two measures. The area under the ROC curve (AUC) was 0.743 for mean LA voltage and 0.717 for LVA%, with no significant difference between the two (bootstrap test for correlated ROC curves, *p =* 0.392) (Figure 5). At the optimal cut-off value of 0.72 mV, mean LA voltage demonstrated a sensitivity of 72% and specificity of 70% for identifying prior ischemic stroke. For LVA%, the optimal threshold of 42.2% yielded a sensitivity of 76% and specificity of 62%.

In model performance comparisons, the mean voltage-only model showed better overall fit than the LVA%-only model, with a lower AIC (166.23 vs. 171.05) and higher pseudo-R^2^ (Nagelkerke 0.143 vs. 0.109). In nested model comparisons, adding LVA% to the mean voltage-only model did not significantly improve model fit (likelihood ratio test *p =* 0.620), whereas adding mean LA voltage to the LVA%-only model significantly improved model fit (*p =* 0.024).

## 4. Discussion

This study revealed that AF patients with a prior stroke have a significantly lower mean LA voltage and greater LVA%. In a multivariable analysis incorporating the individual components of the CHA_2_DS_2_-VASc score, only mean LA voltage showed a consistent and independent association with prior stroke. Although the overall discriminative performance of mean LA voltage and LVA% was comparable, adding mean LA voltage to the LVA%-only model improved model fit, whereas the reverse did not.

Atrial cardiomyopathy is increasingly recognized as a fundamental substrate of AF and powerful risk factor for thromboembolic events. It refers to not only structural but also electrophysiological changes in the LA that predispose to arrhythmogenesis and a decrease in hemodynamic function [19]. Among the various markers of atrial cardiomyopathy, LA voltage is emerging as a useful tool to characterize atrial tissue health and to reflect clinically relevant atrial remodeling.

Decreased voltage areas in the LA, often referred to as LVAs, represent zones of impaired conduction and reduced viable myocardium, and their extent correlates with the severity of atrial remodeling and the likelihood of arrhythmia recurrence after ablation [20,21]. Histological studies have confirmed that such regions contain fibrosis, myofibrillar loss, and increased intercellular space [21]. These changes disrupt uniform propagation of electrical signals and reduce atrial contractility, thereby promoting both re-entry and blood stasis with consequent thrombus formation [22]. Voltage mapping therefore provides a window into the severity of atrial cardiomyopathy, and a recent meta-analysis has shown that LVAs are strongly associated with clinical and echocardiographic markers of atrial remodeling [23].

Most prior investigations have focused primarily on the extent of LVA as a marker of atrial remodeling. However, atrial cardiomyopathy is increasingly recognized as a diffuse and progressive process rather than a purely regional abnormality [24]. Prior studies have shown that localized LVAs are accompanied by widespread atrial voltage reduction and slowed conduction, and that LVA-targeted ablation alone has limited efficacy, supporting the concept that atrial remodeling often extends beyond discrete low-voltage regions [25,26,27].

LVA% quantifies the extent of tissue below a predefined voltage threshold and primarily reflects the regional burden of low-voltage myocardium. It may overlook gradations of voltage reduction outside these regions [25]. Mean LA voltage, as a continuous global measure, incorporates information from the entire atrial surface and may therefore more comprehensively reflect the overall atrial substrate. Although the two measures were strongly correlated, their relationship was markedly nonlinear, with LVA% rising steeply as mean LA voltage fell below approximately 0.94 mV and changing only gradually above this value. In the higher voltage range, tissue that LVA% classifies as uniformly normal may still show a wide range of voltages. This gradation is not biologically silent: histological studies have demonstrated diffuse interstitial fibrosis extending well beyond discrete low-voltage regions, and localized LVAs are accompanied by electrophysiological degeneration of the entire left atrium [13,25]. Consistent with this, adding mean LA voltage to the LVA%-only model improved model fit, whereas the reverse did not, suggesting that voltage information above the 0.5 mV threshold carries additional signals relevant to prior stroke. Its practical advantages are that it is continuous, simple to derive, and independent of an arbitrary voltage cut-off, rather than that it discriminates better.

In the context of established stroke risk tools, the CHA_2_DS_2_-VASc score remains the most validated and widely used for estimating thromboembolic risk in patients with atrial fibrillation [6]. In addition to these clinical factors, other markers such as an enlarged LA, elevated natriuretic peptide levels, non-paroxysmal AF, and a non-chicken wing LAA have been linked to increased stroke risk [28,29]. Among these factors, age, LA diameter, type of AF, and NT-pro-BNP showed significant association with mean LA voltage and age was significantly higher in patients with prior stroke in our study. However, in the multivariable model incorporating key components of the CHA_2_DS_2_-VASc score, only mean LA voltage remained independently associated with prior ischemic stroke. It is possible that the association between traditional risk factors for ischemic stroke can be mediated by progressive atrial structural and electrophysiological remodeling. For example, age is a powerful risk factor for ischemic stroke and is also strongly associated with mean LA voltage, with older patients having significantly lower LA voltage [4]. Taken together, mean LA voltage may reflect the cumulative burden of atrial remodeling associated with multiple clinical risk factors, rather than merely serving as an isolated electrophysiological parameter. These findings further support the concept that atrial substrate assessment may provide complementary mechanistic insight into cardioembolic risk. Despite its strong association with thromboembolism, LA voltage cannot be measured by a non-invasive method. This can only be achieved during an AF ablation procedure which is invasive and expensive. Estimation of LA voltage by 12-lead surface electrocardiography, which is non-invasive and relatively cheap, may not be feasible since the LA is located more distally from the chest wall surface than the RA. Whether machine-learning approaches applied to the surface electrocardiogram could estimate the atrial substrate non-invasively remains speculative and would require dedicated validation. Cardiac magnetic resonance imaging is another candidate to evaluate and quantify atrial cardiomyopathy but there is no consensus on the diagnostic criteria and the protocols are not standardized [30]. In addition, whether areas of late gadolinium enhancement do actually have histologic degeneration needs further validation.

The characteristics of the index stroke events provide additional context for these findings. The large majority of patients were not receiving oral anticoagulation at the time of stroke, largely because AF had not yet been diagnosed or was identified only at the time of the cerebrovascular event. Following the diagnosis of AF, all patients were maintained on direct oral anticoagulants up to the index procedure. Differential anticoagulant exposure is therefore unlikely to account for the observed association between mean LA voltage and prior stroke.

Several limitations should be considered. First, the cross-sectional design precludes causal inference; lower LA voltage cannot be interpreted as a predictor of stroke, and stroke itself may promote atrial remodeling. Prospective studies are warranted. Second, the small number of events limited statistical power, and the absence of independent associations for conventional risk factors should not be interpreted as evidence that these factors are unimportant. Firth’s penalized likelihood was applied to mitigate small-sample bias, and the association of mean LA voltage with prior stroke was consistent across univariable, multivariable, and sensitivity models. Third, voltages acquired during AF are systematically lower than those during sinus rhythm, and AF-rhythm mapping was numerically more frequent in the prior-stroke group. The association was unchanged after adjustment for mapping rhythm and persisted in an analysis restricted to patients mapped during sinus rhythm, suggesting that rhythm-related voltage attenuation does not account for the observed findings; residual confounding nonetheless cannot be entirely excluded. Fourth, measurements were obtained by a single operator at a single center, which limits generalizability, although standardized sequential acquisition minimizes variability related to catheter contact and mapping system. Fifth, the 0.94 mV breakpoint and the 0.72 mV ROC-derived threshold were derived from this cohort without internal validation and should be regarded as exploratory rather than as clinically applicable cut-offs. Finally, the prior-stroke endpoint remained heterogeneous. The date of the index stroke was unavailable in seven patients and the subtype could not be classified in eight.

## 5. Conclusions

AF patients with prior ischemic stroke had significantly lower mean LA voltage and higher LVA%. In multivariable analysis incorporating established clinical risk factors, only mean LA voltage remained independently associated with prior stroke. Compared with LVA%, mean LA voltage may better reflect the underlying atrial substrate, supporting its potential role as a continuous, threshold-independent measure of atrial remodeling in patients undergoing AF ablation.

## Figures and Tables

**Table 1:** Baseline characteristics.

Variables	Total (*n* = 259)	Stroke (*n* = 29)	Without Stroke (*n* = 230)	*p*-Value
Age, years	67.0 [58.0–74.0]	72.0 [63.0–77.0]	67.0 [58.0–74.0]	0.033
Male, *n* (%)	177 (68.3%)	19 (65.5%)	158 (68.7%)	0.893
BMI, kg/m^2^	25.4 [23.3–27.6]	25.0 [22.8–25.8]	25.5 [23.3–27.6]	0.290
Hypertension, *n* (%)	167 (64.5%)	18 (62.1%)	149 (64.8%)	0.935
Diabetes, *n* (%)	64 (24.7%)	6 (20.7%)	58 (25.2%)	0.761
Dyslipidemia, *n* (%)	119 (45.9%)	17 (58.6%)	102 (44.3%)	0.209
Heart failure, *n* (%)	30 (11.6%)	2 (6.9%)	28 (12.2%)	0.547
Vascular disease, *n* (%)	22 (8.5%)	3 (10.3%)	19 (8.3%)	0.722
CHA_2_DS_2_-VASc score	2.0 [1.0–3.0]	5.0 [3.0–5.0]	2.0 [1.0–3.0]	<0.001
Persistent atrial fibrillation, *n* (%)	159 (61.4%)	18 (62.1%)	141 (61.3%)	>0.99
Hemoglobin, g/dL	14.2 ± 1.6	13.3 ± 1.9	14.3 ± 1.6	0.008
Platelet count, ×10^3^/μL	211.0 [183.0–243.0]	186.0 [169.0–221.0]	214.5 [188.0–247.0]	0.004
eGFR, mL/min/1.73 m^2^	74.0 ± 21.2	71.0 ± 27.8	74.4 ± 20.2	0.539
HbA1c, %	5.7 [5.4–6.2]	5.7 [5.4–5.9]	5.7 [5.4–6.2]	0.331
NT-proBNP, pg/mL	468.5 [135.5–963.5]	546.0 [300.0–1103.0]	467.0 [133.0–941.0]	0.057
LA diameter, mm	42.7 ± 6.3	43.9 ± 5.1	42.6 ± 6.4	0.235
Atrial fibrillation during mapping, *n* (%)	27 (10.4%)	6 (20.7%)	21 (9.1%)	0.097
Number of mapped points	2104 [1757–2491]	2083.0 [1872–2439]	2108.0 [1756–2507]	0.879
Mean LA voltage, mV	1.0 [0.6–1.3]	0.5 [0.4–0.8]	1.0 [0.6–1.3]	<0.001
Low-voltage point proportion, %	50.1 [39.5–62.7]	66.9 [53.5–76.2]	47.8 [39.1–60.1]	<0.001
LVA%, %	37.3 [27.1–55.4]	60.2 [42.3–69.2]	36.5 [26.4–51.1]	<0.001

Continuous variables are presented as mean ± SD or median [IQR], and categorical variables as counts and percentages. Abbreviations: AF, atrial fibrillation; BMI, body mass index; eGFR, estimated glomerular filtration rate; HbA1c, glycated hemoglobin; LA, left atrium/left atrial; LVA%, low-voltage area percentage; NT-proBNP, N-terminal pro B-type natriuretic peptide.

**Table 2:** Logistic regression analyses for factors associated with prior ischemic stroke.

	Univariable		Multivariable	
Variable	OR (95% CI)	*p*-Value	OR (95% CI)	*p*-Value
Age, years	1.046 (1.007–1.091)	0.019	1.011 (0.964–1.064)	0.651
Male	0.849 (0.388–1.953)	0.691	1.352 (0.579–3.313)	0.491
BMI, kg/m^2^	0.955 (0.847–1.065)	0.418		
Hypertension	0.877 (0.405–1.971)	0.744	0.884 (0.389–2.076)	0.771
Diabetes	0.816 (0.301–1.942)	0.659	0.732 (0.262–1.814)	0.514
Dyslipidemia	1.755 (0.817–3.871)	0.149		
Heart failure	0.646 (0.126–2.120)	0.506	0.474 (0.089–1.651)	0.263
Vascular disease	1.018 (0.471–2.283)	0.965	1.238 (0.297–4.036)	0.746
Persistent atrial fibrillation	1.433 (0.361–4.331)	0.572		
Left atrial diameter, mm	1.034 (0.968–1.105)	0.322		
NT-proBNP, per 100 pg/mL	1.012 (0.974–1.039)	0.423		
Mean LA voltage, mV	0.111 (0.033–0.323)	<0.001	0.117 (0.032–0.381)	<0.001
LVA%, %	1.037 (1.018–1.057)	<0.001		

Univariable and multivariable logistic regression analyses were performed using Firth’s penalized logistic regression to account for the small number of events and potential separation. The multivariable model included age, sex, hypertension, diabetes mellitus, heart failure, vascular disease, and mean left atrial voltage. Abbreviations: BMI, body mass index; CI, confidence interval; LA, left atrial; LVA%, low-voltage area percentage; NT-proBNP, N-terminal pro–B-type natriuretic peptide; OR, odds ratio.

## Data Availability

The data presented in this study are available on request from the corresponding author due to privacy and ethical restrictions.

## Supplementary Materials

The following supporting information can be downloaded at https://www.mdpi.com/article/10.3390/jcm15187036/s1, Table S1: Sensitivity analyses for the association between mean left atrial voltage and prior ischemic stroke. Table S2: Multivariable analysis restricted to patients mapped during sinus rhythm.

## Abbreviations

The following abbreviations are used in this manuscript:

AF	Atrial fibrillation
AUC	Area under the curve
LA	Left atrium/left atrial
LAA	Left atrial appendage
LVA%	Low-voltage area percentage
NT-proBNP	N-terminal pro-B-type natriuretic peptide
RFCA	Radiofrequency catheter ablation
ROC	Receiver operating characteristic
