# Supplementary material for: Association Between Mean Left Atrial Voltage and Prior Ischemic Stroke in Atrial Fibrillation: A Single-Center Cross-Sectional Study
Source: J Clin Med. 2026 Sep 11;15(18):7036. doi: 10.3390/jcm15187036 (PMC13607983; doi:10.3390/jcm15187036)
Supplement: Supplementary file 1 [file jcm-15-07036-s001.zip › jcm-4508430-supplementary.pdf]

Association Between Mean Left Atrial Voltage and Prior Ischemic Stroke in Atrial Fibrillation: A Single-Center Cross-sectional Study

Supplementary material

Table S1. Sensitivity analyses for the association between mean left atrial voltage and prior ischemic stroke

Variable	Multivariable OR (95% CI)	*P*-value
Age, years	1.007 (0.959–1.062)	0.776
Male	1.329 (0.569–3.254)	0.516
Hypertension	0.835 (0.361–1.983)	0.676
Diabetes	0.764 (0.272–1.913)	0.578
Heart failure	0.474 (0.082–1.798)	0.296
Vascular disease	1.083 (0.255–3.588)	0.904
Mean LA voltage, mV	0.061 (0.012–0.264)	<0.001
Atrial fibrillation during mapping	1.178 (0.319–4.220)	0.802
Number of mapped points, per 100	0.907 (0.827–0.989)	0.026

Multivariable Firth logistic regression model additionally adjusts for mapping rhythm and the number of mapped points to account for potential confounding by mapping density. Abbreviations: CI, confidence interval; LA, left atrial; OR, odds ratio.

Table S2. Multivariable analysis restricted to patients mapped during sinus rhythm

	Multivariable	
**Variable**	**OR (95% CI)**	***P*-value**
Age, years	1.001 (0.947–1.060)	0.980
Male	1.402 (0.541–3.868)	0.492
Hypertension	0.810 (0.332–2.051)	0.648
Diabetes	1.101 (0.374–2.955)	0.853
Heart failure	0.540 (0.101–1.913)	0.366
Vascular disease	1.468 (0.344–4.952)	0.573
Mean LA voltage, mV	0.090 (0.017–0.398)	0.001

Multivariable Firth logistic regression restricted to patients in whom electroanatomic mapping was performed during sinus rhythm (n = 232; 23 events). Abbreviations: CI, confidence interval; LA, left atrial; OR, odds ratio.
